# Geographical Heterogeneity of Multiple Sclerosis Prevalence in France

**DOI:** 10.1371/journal.pone.0167556

**Published:** 2016-12-09

**Authors:** Diane Pivot, Marc Debouverie, Michel Grzebyk, David Brassat, Michel Clanet, Pierre Clavelou, Christian Confavreux, Gilles Edan, Emmanuelle Leray, Thibault Moreau, Sandra Vukusic, Guy Hédelin, Francis Guillemin

**Affiliations:** 1 Clinical and Evaluation department, Nancy University Hospital, Vandoeuvre-les-Nancy, France; 2 EA 4360 Apemac, Lorraine University, Paris Descartes University, Nancy, France; 3 Department of Neurology, Nancy University Hospital, Nancy, France; 4 National Institute for Occupational Safety and Health, Vandoeuvre-lès-Nancy, France; 5 Department of Neurology, Toulouse University Hospital, Toulouse, France; 6 Department of Neurology, Clermont-Ferrand University Hospital, Clermont-Ferrand, France; 7 Department of Neurology, Lyon Hospices Civils - Pierre Wertheimer University Hospital, Lyon, France; 8 Department of Neurology, Rennes University Hospital, Rennes, France; 9 Ecoles des Hautes Etudes en Santé Publique, Rennes, France; 10 Department of Neurology, Dijon University Hospital, Dijon, France; 11 INSERM, CIC-EC, Nancy, France; Taipei Veterans General Hospital, TAIWAN

## Abstract

**Introduction:**

Geographical variation in the prevalence of multiple sclerosis (MS) is controversial. Heterogeneity is important to acknowledge to adapt the provision of care within the healthcare system. We aimed to investigate differences in prevalence of MS in departments in the French territory.

**Methods:**

We estimated MS prevalence on October 31, 2004 in 21 administrative departments in France (22% of the metropolitan departments) by using multiple data sources: the main French health insurance systems, neurologist networks devoted to MS and the Technical Information Agency of Hospitalization. We used a spatial Bayesian approach based on estimating the number of MS cases from 2005 and 2008 capture–recapture studies to analyze differences in prevalence.

**Results:**

The age- and sex-standardized prevalence of MS per 100,000 inhabitants ranged from 68.1 (95% credible interval 54.6, 84.4) in Hautes-Pyrénées (southwest France) to 296.5 (258.8, 338.9) in Moselle (northeast France). The greatest prevalence was in the northeast departments, and the other departments showed great variability.

**Discussion:**

By combining multiple data sources into a spatial Bayesian model, we found heterogeneity in MS prevalence among the 21 departments of France, some with higher prevalence than anticipated from previous publications. No clear explanation related to health insurance coverage and hospital facilities can be advanced. Population migration, socioeconomic status of the population studied and environmental effects are suspected.

## Introduction

Determining the prevalence of multiple sclerosis (MS) is important for assessing the burden of this disease in the population and to society. At the national level, a heterogeneous distribution of cases over a territory would require organizing an adequate distribution of healthcare resources. Moreover, demonstrating geographical variation in prevalence would suggest new avenues for research to further explore spatial or environmental hypotheses [1].

The prevalence of MS is not homogenous in the world [2–5]. It varies greatly between northern and southern countries [6]. There are gradients at the country level [6]: the prevalence increases from south to north in Japan [2] and Europe [3], and from north to south in Australia [4] and South America [5]. Despite these variations in many geographical areas, the association of prevalence and latitude is contested by several studies [7–13]. Such comparisons are limited by the heterogeneity of the diagnostic criteria used for selecting cases, population characteristics, geographical scale, methodological design and statistical methods. Thus, the notion of a gradient could be due to methodological artifacts.

People in France, located in the middle latitude of Western Europe, are considered to be at medium to high risk for MS [14]. Recent studies have shown variability in space in MS prevalence, with prevalence ranging from 110 per 100,000 inhabitants in the southwest part of the country [15] to 188.2 per 100,000 in the northeast [16]. The variation in MS prevalence could be explained by population migration, which can lead to modification in spatial repartition of MS susceptibility genes [7].

Use of spatial analysis with different geographical scales may provide different types of information [17,18]. The first study of geographical variation in MS prevalence performed in France in 2003 was based on a subset of 7% of the French population covered by the national health insurance system for farmers and revealed a decreasing northeast to southwest gradient on a regional scale [3]. A second study was conducted in 2004 with a much larger and representative subset of 87% of the French population insured by the general national health insurance system [13]. The analysis of these data with a Bayesian method suggested a heterogeneous distribution of MS prevalence across the administrative departments in France rather than a true geographical gradient. Furthermore, two capture–recapture studies were conducted in Haute-Garonne, located in the southwestern part of France [15], and in the four administrative departments of the northeastern Lorraine region in 2005 and 2008 [16]. As compared with previous studies, these studies revealed increased prevalence in these five departments. More recently, a study of a population of independent workers that involved a Bayesian method found a decreasing northeast to southwest gradient on departmental scale [19].

The aim of our study, conducted as part of the French Multiple Sclerosis Observatory (Observatoire Français de la Sclérose en Plaques, OFSEP) initiative, was to investigate differences in MS prevalence in administrative departments in France with a spatial Bayesian model. The data are from multiple sources, including the two capture–recapture studies, in multiple regions over the French territory.

## Methods

### Design and setting

**Fig 1:**
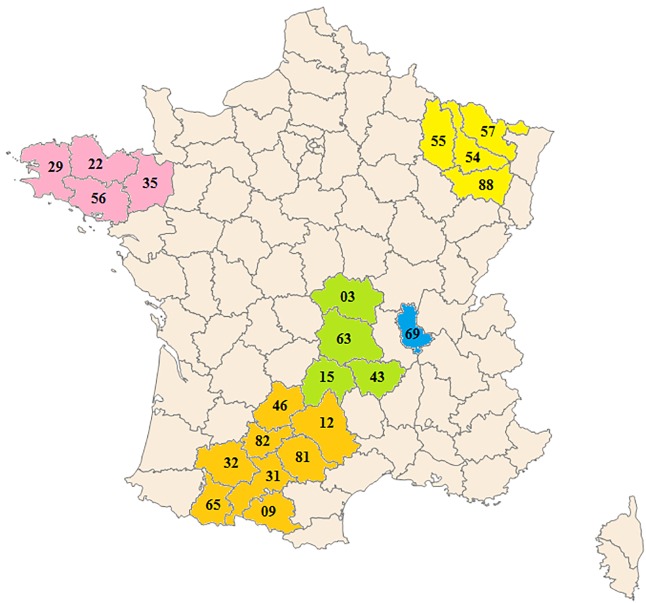
Administrative departments in France (coding number) ranked by latitude. Yellow: **Northeast region (NE) Lorraine**: Moselle (57); Meuse (55); Meurthe-et-Moselle (54); Vosges (88) Pink: **Northwest region (NW) Bretagne**: Côtes-d’Armor (22); Finistère (29); Ille-et-Vilaine (35); Morbihan (56) Green**: Center region (C) Auvergne**: Allier (03); Haute-Loire (43); Puy-de-Dôme (63); Cantal (15) Blue**: Southeast region (SE) Rhône-Alpes**: Rhône (69) Orange**: Southwest region (SW) Midi-Pyrénées**: Lot (46); Aveyron (12); Tarn-et-Garonne (82); Tarn (81); Gers (32); Haute-Garonne (31); Hautes-Pyrénées (65); Ariège (09).

This multisource epidemiological cross-sectional descriptive study was of prevalent cases of MS alive on October 31, 2004, in 21 French administrative departments in the national territory in France (Fig 1).

France comprises 96 administrative areas called departments. These departments are grouped in 22 regions, with a region typically consisting of 2 to 8 departments.

These geographical areas were selected by two criteria: resident neurologists 1) established a long-standing network devoted to regional and departmental ambulatory and hospital care of MS patients and 2) used the European Database for Multiple Sclerosis software in daily practice as a medical file allowing uniform data collection [20].

### MS cases definition

The target population of cases for this study consisted of all people with a diagnosis of MS, whatever the date of recognition as a long-term disease before October 31, 2004, their age, gender or clinical course, who resided in one of the departments at the time of study.

The national CNIL ethics committee approved this study (CNIL nos. 1641449 and 1641449v1).

### Calculation of observed number of cases of MS

We calculated the number of MS cases by department, sex and age from a combination of three data sources, namely neurologist networks, health insurance systems and hospital information agency, using the share of overlap between sources derived from the previous capture–recapture studies [15,16] (see S1 Methods).

The Registre Lorrain de la Sclérose en Plaques (ReLSEP) has a unique characteristic in France and Europe [16] of providing data on geographic prevalence and incidence by crossing cases of MS in the Lorraine region. ReLSEP uses the same three sources of case records as used in the present study but restricted to Lorraine (NE).

ReLSEP performed a capture–recapture study with data for 2008 [16], in which data issued from these three sources were used to obtain shares of overlap at the regional and the departmental level by sex and age class. In the present study, we applied those shares of overlap to combine the three sources in all departments studied and estimate the number of unique cases of MS.

This number of cases represented the numerator of departmental prevalence.

These calculations were performed under the assumption of homogeneity of shares of overlap between sources whatever the geographical location of departments and the assumption of stability in prevalence between 2004 and 2008.

### Population

We obtained the total population in each department for the year 2004 from the national census at the National Institute of Statistical and Economic Information (INSEE, 2013). This number formed the denominator for departmental prevalence of MS.

### Statistical analysis

First, we tested spatial autocorrelation between neighbouring departments, defined as departments sharing a common border. This phenomenon can be identified by using the Moran test. The Moran index summarizes the degree of similarity of neighbouring geographical units with a weighted average of the similarity between observations. We computed the standardized prevalence ratio for each department (i.e., total number of observed to expected cases × 100). Then, we computed the Moran index based on the expected number of MS cases by age and sex in each department by an internal indirect standardized method.

Second, to compare the MS prevalence between departments adjusted for sex and age, we used a Bayesian model with a binomial negative distribution to allow for overdispersion. We chose a conditional autoregressive (CAR) model to account for spatial autocorrelation. This model has a global structure that is used to compare the prevalence between each department adjusted for sex and age and a local structure that accounts for the spatial autocorrelation that can exist between neighboring departments [21]. Non-informative priors were used for the Bayesian model. From these priors and the computed number of MS cases in each department, age class and sex, the model was estimated by Monte Carlo Markov Chain sampling techniques with which prevalence and relative risk for each department were derived. The precision of these estimates is given with 95% credible intervals (95% CrIs). Finally, we performed direct standardization using the French reference population as defined by INSEE to reflect the structure of the French population and using the European and the world reference population (World Health Organization) for international comparison.

### Sensitivity analysis

To test the robustness of the model, we performed a sensitivity analysis replacing the Lorraine region with the departmental shares of overlap estimated from capture–recapture studies in each of the four Lorraine departments (NE) [16] and the Haute-Garonne department (SW) [15] consecutively.

### Data analysis software

We used Microsoft Excel 2010 to calculate the number of expected cases, the standardized prevalence ratio and the Moran index. SAS 9.3 (SAS Inst., Cary, NC) was used for data management and production of maps. Finally, Winbugs 1.4 was used to compute the number of unique MS cases, fit the conditional autoregressive model, and estimate the relative risks and the crude and standardized prevalence.

## Results

### Description of cases

On October 31, 2004, the main French health insurance systems (CNAMTS and MSA) recorded 2,833 patients with MS, the MS network 3,462 patients and the ATIH 4,295 patients in Lorraine (NE); 2,678, 1,698 and 3,529, respectively, in Bretagne (NW); 1,365, 583 and 965 in Auvergne (C); 1,429, 1,932 and 1,237 in Rhône (SE) and 2,226, 2,068 and 2,806 in Midi-Pyrénées (SW) (Table 1).

**Table 1:** Number of multiple sclerosis (MS) cases obtained from each data source for the year 2004.

French administrative region with their departments	Health insurance systems	Neurologist network	Hospitalization	No. of unique observed cases ^a^
**Northeast region**	**2,833**	**3,462**	**4,295**	**5,961**
Moselle	1,298	1,161	2,507	3036
Meuse	244	296	226	395
Meurthe-et-Moselle	918	1,425	1,112	1799
Vosges	373	580	450	731
**Northwest region**	**2,678**	**1,698**	**3,529**	**4,387**
Côtes-d’Armor	579	258	868	975
Finistère	837	370	961	1203
Ille-et-Vilaine	751	717	904	1275
Morbihan	511	353	796	934
**Center region**	**1,365**	**583**	**965**	**1,539**
Allier	413	84	398	498
Haute-Loire	209	39	148	214
Puy-de-Dôme	591	373	301	638
Cantal	152	87	118	189
**Southeast region**	**1,429**	**1,932**	**1,237**	**2,336**
**Southwest region**	**2,226**	**2,068**	**2,806**	**3,790**
Lot	146	116	183	244
Aveyron	240	141	257	347
Tarn-et-Garonne	167	133	145	232
Tarn	306	259	418	539
Gers	156	125	177	247
Haute-Garonne	940	1,054	1,420	1,812
Hautes-Pyrénées	152	120	21	136
Ariège	119	120	185	233

^a^ The number of unique observed cases was calculated with 0.25 share of overlap between the health insurance systems and neurologist network, 0.28 between the neurologist network and hospitalization, 0.42 between the health insurance system and hospitalization, and 0.17 between the three sources for the department of Haute-Garonne based on the capture–recapture study of 2005 [15]. For the other departments, the calculated shares were 0.46, 0.23, 0.20 and 0.12, respectively, based on the capture–recapture study of 2008 [16].

### Prevalence

We revealed a spatial autocorrelation between neighbouring departments by the Moran index (Z = 3, 06; p = 0.002). Thus, we accounted for the local structure in the Bayesian spatial model, which revealed an estimated mean prevalence of MS of 167.4/100,000 inhabitants in the 21 departments under study (95% CrI 159.0, 176.5).

**Fig 2:**
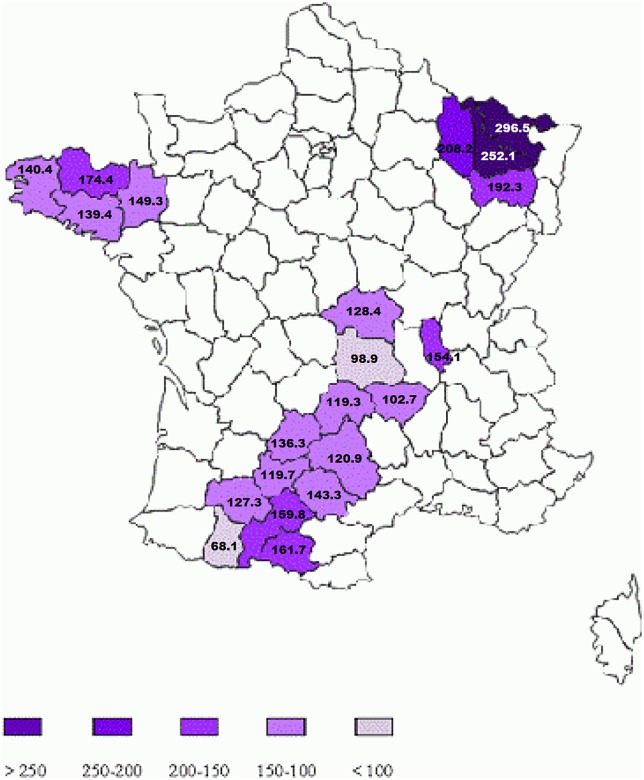
Standardized MS prevalence per 100,000 inhabitants for the 21 departments under study.

The standardized prevalence per 100,000 inhabitants ranged from 68.1 (95% CrI 54.6, 84.4) in Hautes-Pyrénées (SW) to 296.5 (258.8, 338.9) in Moselle (NE) (Table 2). The four Lorraine departments (NE) showed the highest prevalence, from 192.3 (165.4, 223.2) in Vosges to 296.5 (258.8, 338.9) in Moselle (Table 2; Fig 2). The departments in the Auvergne (C) and Midi-Pyrénées (SW) regions showed a lower prevalence.

**Table 2:** Estimated age- and sex-standardized MS prevalence per 100,000 inhabitants (French population).

Administrative department of France	MS standardized prevalence ^a^	95% CrI ^b^
Moselle (NE)	296.5	258.8–338.9
Meurthe-et-Moselle (NE)	252.1	220.4–287.4
Meuse (NE)	208.2	176.7–244.0
Vosges (NE)	192.3	165.4–223.2
Côtes-d'Armor (NW)	174.4	150.9–200.8
Ariège (SW)	161.7	134.4–193.2
Haute-Garonne (SW)	159.8	139.4–182.9
Rhône (SE)	154.1	134.2–176.1
Ille-et-Vilaine (NW)	149.3	129.3–171.7
Tarn (SW)	143.3	123.0–166.0
Finistère (NW)	140.4	121.7–161.3
Morbihan (NW)	139.4	120.7–161.1
Lot (SW)	136.3	115.3–160.6
Allier (C)	128.4	109.3–150.0
Gers (SW)	127.3	106.3–151.3
Aveyron (SW)	120.9	102.9–141.1
Tarn-et-Garonne (SW)	119.7	100.6–141.0
Cantal (C)	119.3	100.1–141.8
Haute-Loire (C)	102.7	85.7–121.8
Puy-de-Dôme (C)	98.9	84.8–114.3
Hautes-Pyrénées (SW)	68.1	54.6–84.4

^a^ The prevalence was calculated by using 0.25 of overlap between the health insurance system and neurologist network, 0.28 between the neurologist network and hospitalization, 0.42 between the health insurance system and hospitalization, and 0.17 between the three sources for the department of Haute-Garonne based on the capture–recapture study of 2005 [15]. For the other departments, the calculated shares were 0.46, 0.20, 0.23 and 0.12, respectively, based on the capture–recapture study of 2008 [16].

^b^ 95% CrI, 95% credible interval.

The same trends were observed with the European and world-standardized prevalence per 100,000 inhabitants. Indeed, the ranking of prevalence did not differ by the standardized population used (Table 3).

**Table 3:** Estimation of age- and sex-standardized MS prevalence ^a^ per 100,000 inhabitants in European and world populations.

Administrative department of France	European standardizedprevalence	95% CrI ^a^	World standardized prevalence	95% CrI ^b^
Moselle (NE)	292.2	255.0–334.0	264.9	231.1–303.0
Meurthe-et-Moselle (NE)	248.4	217.1–283.8	225.3	196.8–257.1
Meuse (NE)	205.2	174.1–240.5	186.1	157.8–218.1
Vosges (NE)	189.5	162.9–220.0	171.8	147.7–199.5
Côtes-d'Armor (NW)	171.9	148.7–197.9	155.8	134.8–179.4
Ariège (SW)	159.3	132.4–190.4	144.5	120.1–172.6
Haute-Garonne (SW)	157.4	137.4–180.2	142.7	124.5–163.5
Rhône (SE)	151.9	132.2–173.6	137.7	119.8–157.4
Ille-et-Vilaine (NW)	147.1	127.4–169.2	133.4	115.6–153.4
Tarn (SW)	141.2	121.2–163.6	128.0	109.9–148.4
Finistère (NW)	138.3	119.9–158.9	125.4	108.8–144.1
Morbihan (NW)	137.3	118.9–158.8	124.5	107.8–143.9
Lot (SW)	134.3	113.6–158.2	121.8	103.0–143.5
Allier (C)	126.5	107.7–147.7	114.7	97.6–134.1
Gers (SW)	125.4	104.8–149.1	113.7	95.0–135.3
Aveyron (SW)	119.1	101.3–139.0	108.0	91.9–126.1
Tarn-et-Garonne (SW)	117.9	99.16–138.9	106.9	89.9–125.9
Cantal (C)	117.5	98.58–139.7	106.6	89.3–126.7
Haute-Loire (C)	101.1	84.5–120.0	91.7	76.6–108.8
Puy-de-Dôme (C)	97.4	83.6–112.6	88.3	75.7–102.1
Hautes-Pyrénées (SW)	67.1	53.8–83.2	60.9	48.7–75.4

^a^ on October 31, 2004,

^b^ 95% credible interval.

The magnitude of these trends was highlighted when considering the relative risk of MS for each department as compared with the average for the 21 departments. The four Lorraine departments (NE) and Côtes-d’Armor (NW) showed high risk with reference to the mean of the overall departments studied (Table 4). The relative risk varied from 1.2 (95% CrI 1.1, 1.4) for Côtes-d’Armor (NW) to 2.1 (1.8, 2.3) for Moselle (NE). Three departments had a low risk, with relative risk ranging from 0.5 (0.4, 0.6) for Hautes-Pyrénées (SW) to 0.7 (0.6, 0.8) for Haute-Loire (C).

**Table 4:** Relative risk of MS for each department compared to the mean prevalence for all departments ^a^.

Administrative department of France	Relative risk	95% CrI ^b^
Moselle (NE)	2.1	1.8–2.3
Meurthe-et-Moselle (NE)	1.7	1.5–2.0
Meuse (NE)	1.4	1.2–1.7
Vosges (NE)	1.3	1.2–1.5
Côtes-d'Armor (NW)	1.2	1.1–1.4
Ariège (SW)	1.1	0.9–1.3
Haute-Garonne (SW)	1.1	1.0–1.3
Rhône (SE)	1.1	0.9–1.2
Ille-et-Vilaine (NW)	1.0	0.9–1.2
Tarn (SW)	1.0	0.9–1.1
Finistère (NW)	1.0	0.8–1.1
Morbihan (NW)	1.0	0.8–1.1
Lot (SW)	0.9	0.8–1.1
Allier (C)	0.9	0.8–1.0
Gers (SW)	0.9	0.7–1.0
Aveyron (SW)	0.8	0.7–1.0
Tarn-et-Garonne (SW)	0.8	0.7–1.0
Cantal (C)	0.8	0.7–1.0
Haute-Loire (C)	0.7	0.6–0.8
Puy-de-Dôme (C)	0.7	0.6–0.8
Hautes-Pyrénées (SW)	0.5	0.4–0.6

^a^ with reference to the Lorraine shares of overlap, on 31 October, 2004

^b^ 95% credible interval.

### Sensitivity analysis

Using the shares of overlap for Haute-Garonne (SW), considered the highest, the same trends were observed for prevalence and relative risk, with only slight differences in the ranking of departments. The four Lorraine departments (NE) and the Rhône department (SE) had the highest prevalence (Table 5). The same departments as in the main analysis had the lowest prevalence. The same trends were also observed when using the shares of overlap for Moselle (NE), considered the lowest (Table 6).

**Table 5:** Relative risk using the department with the highest shares of overlap, Haute-Garonne^a^.

Administrative department of France	Relative risk	95% CrI ^b^
Moselle (NE)	2.2	1.9–2.5
Meurthe-et-Moselle (NE)	1.7	1.5–1.9
Vosges (NE)	1.3	1.2–1.5
Meuse (NE)	1.3	1.1–1.5
Rhône (SE)	1.2	1.1–1.3
Haute-Garonne (SW)	1.1	1.0–1.3
Côtes-d'Armor (NW)	1.1	1.0–1.2
Ariège (SW)	1.1	0.9–1.3
Ille-et-Vilaine (NW)	1.0	0.9–1.2
Tarn (SW)	1.0	0.9–1.1
Lot (SW)	0.9	0.8–1.1
Finistère (NW)	0.9	0.8–1.0
Morbihan (NW)	0.9	0.8–1.0
Gers (SW)	0.9	0.8–1.0
Tarn-et-Garonne (SW)	0.9	0.7–1.0
Allier (C)	0.8	0.7–1.0
Aveyron (SW)	0.8	0.7–1.0
Cantal (C)	0.8	0.7–1.0
Puy-de-Dôme (C)	0.7	0.6–0.8
Haute-Loire (C)	0.7	0.6–0.8
Hautes-Pyrénées (SW)	0.6	0.5–0.7

^a^ with the average prevalence for the 21 departments as a reference.

^b^ 95% credible interval.

**Table 6:** Relative risk using the department with the lowest shares of overlap, Moselle ^a^.

Administrative department of France	Relative risk	95% CrI ^b^
Moselle (NE)	2.0	1.8–2.3
Meurthe-et-Moselle (NE)	1.6	1.4–1.8
Vosges (NE)	1.3	1.1–1.4
Meuse (NE)	1.2	1.0–1.4
Côtes-d'Armor (NW)	1.2	1.0–1.4
Ariège (SW)	1.1	0.9–1.3
Rhône (SE)	1.1	1.0–1.3
Haute-Garonne (SW)	1.1	1.0–1.2
Ille-et-Vilaine (NW)	1.0	0.9–1.2
Tarn (SW)	1.0	0.9–1.1
Finistère (NW)	1.0	0.9–1.1
Morbihan (NW)	1.0	0.8–1.1
Lot (SW)	1.0	0.8–1.1
Allier (C)	0.9	0.8–1.0
Gers (SW)	0.9	0.8–1.1
Tarn-et-Garonne (SW)	0.9	0.7–1.0
Aveyron (SW)	0.9	0.7–1.0
Cantal (C)	0.8	0.7–1.0
Haute-Loire (C)	0.7	0.6–0.9
Puy-de-Dôme (C)	0.7	0.6–0.8
Hautes-Pyrénées (SW)	0.5	0.4–0.6

^a^ with the average prevalence for the 21 departments as a reference.

^b^ 95% credible interval.

## Discussion

### Main findings

Using multiple sources of case identification, we showed a geographical heterogeneity of MS prevalence among 21 administrative departments in France, with the highest standardized prevalence (296.5/100,000) being four times that of the lowest prevalence (68.1/100,000). We found the highest MS standardized prevalence in the Lorraine departments (NE), the Côtes-d’Armor department (NW), 2 SW departments (Ariège and Haute-Garonne) and the Rhône department (SE region). Furthermore, the lowest prevalence was found in the departments of the Auvergne region, located in the center of France. Therefore, these results do not show a clear northeast to southwest gradient among the 21 departments under study, as was previously suggested in the 2003 national study of data for French farmers [3] and more recently among independent workers [19], but the results are closer to those in the 2004 national study of data for the main national health insurance system (CNAMTS) [13]. Moreover, the high prevalence in Haute-Garonne (SW) is consistent with the results obtained in the capture–recapture study conducted in Haute-Garonne in 2005 [15].

Hospital access as well as the provision of care offered to patients can differ by medical center and department. This situation may explain the high prevalence of MS in certain departments. However, our results do not fully account for the observed heterogeneity in prevalence between departments under study. Indeed, the prevalence in Puy-de-Dôme (C) is one of the lowest despite the existence of a university hospital. Thus, it would be pertinent to analyze the relation between provision of care and the prevalence observed.

The particularly high prevalence of MS in some departments could also indicate an environmental risk exposure in these departments. The low level of sunlight and the existence of susceptibility genes and alleles regulated by vitamin D have been suggested as risk factors of MS [1,7,13,22–25]. However, because the prevalence was high in two departments of southwestern France, with a high number of sunlight hours, this set of risk factors is unlikely to be the only cause of MS. Migration of the at-risk population and other risk factors such as infections, by the Epstein-Barr virus, smoking, cultural factors, dietary behavior and income, which is also linked to infections in childhood, have been suggested and should be further explored [22,26]. To better understand this uneven distribution of MS cases, new specific studies should compare departments with high versus low risk.

### Strengths

Previous national studies estimating MS prevalence included only one source of data. The first covered only 7% of the French population [3]. Although the second study increased the accuracy by using a source covering 87% of the population [13], use of only one source of data can lead to an underestimation of MS prevalence in France, as was demonstrated in the two capture–recapture studies [15,16]. We used a new methodology based on the use of multiple sources to improve the quality and comparability of MS prevalence. We included the two main French health insurance systems, which in 2004 covered 90% of the population of the 21 departments studied. Our sensitivity analysis revealed heterogeneity between departments similar to that in the principal analysis. These findings support the robustness of the model. Moreover, the use of the CAR model with a Bayesian approach leads to a geographic smoothing effect. Therefore, different estimated MS prevalences would reinforce the plausibility of the heterogeneity finding.

### Limitations

First, several studies have suggested that the observed heterogeneity in MS distribution could be due to an artifact in methodology [8,10,12,24]. Because obtaining complete data is difficult, the use of incomplete data when calculating MS prevalence may lead to underestimation, even when combining several sources.

Second, the use of share of overlap from the two capture–recapture studies combined with cases of MS identified on October 31, 2004 relied on the hypothesis of a stable overlap over the study period (2004 to 2008). This situation is likely to be the case in a stable healthcare system without changes in medical practices.

Third, we cannot rule out some misclassification in the various sources. With counting by coding, data from the ATIH may have generated another disease motivating the hospital stay. The categorization of MS patients in the CNAMTS and MSA official categories for MS for long-term illness (ALD 25) may vary by practitioners and decision-makers in the national insurance system, with a risk of false-positive and false-negative diagnoses.

Fourth, another limitation of the study is that, because this was not a capture–recapture study of each of the 21 departments, we could not apply a differential share of overlap to each department, thus possibly introducing over- or underestimation of prevalence, without knowing its possibly non-uniform direction or its consequence on the ranking of departments.

So the difference in ranking with other studies showing a north to south gradient should be considered with caution. Some factors such as population migration and socioeconomic status of the population included in the study should be considered. Nevertheless, shares of overlap varied between the four Lorraine departments and the Haute-Garonne department, which could be explained by differing local strategies implemented to care for MS patients in terms of hospitalization. This bias may have blurred the existence of a decreasing northeast to southwest gradient.

## Conclusion

In summary, our results tend to show a geographical heterogeneity in MS distribution in 21 administrative departments in France that is close to previous findings [3,13,15,16], but our prevalence results are much higher than those previously reported. The differences in prevalence between departments may be due to several factors. If we assume that there are not any real differences by the health insurance systems used or the extent of neurologist networks, the effects of population migration, socioeconomic status of the population included and the environment should still be explored. The new methodology combining multiple sources in a spatial Bayesian model provided more accurate estimates of MS prevalence and should be further confirmed with data for more departments in France, taking into account the previously mentioned factors.

## Supporting Information

S1 Methods Medical and administrative data sources. The three medical and administrative data sources are described in this file.
(DOCX)
