# Supplementary material for: Geographical Heterogeneity of Multiple Sclerosis Prevalence in France
Source: PLoS One. 2016 Dec 9;11(12):e0167556. doi: 10.1371/journal.pone.0167556 (PMC5147922; doi:10.1371/journal.pone.0167556)
Supplement: S1 Methods — The three medical and administrative data sources are described in this file. (DOCX) [file pone.0167556.s001.docx]

**Medical and administrative data sources**

First, we asked the regional and departmental ambulatory and hospital care neurologist network in each region to provide the number of cases with a MS diagnosis according to the criteria of McDonald by administrative department. The following criteria were used for selecting MS cases: MS onset satisfying the McDonald criteria before October 31, 2004, and residence in a department under study. Patients who did not satisfy these criteria (although recorded in the regional network) and those who died before October 31, 2004 were not included.

Second, we queried the main French health insurance systems, the Caisse Nationale d’Assurance Maladie des Travailleurs Salariés (CNAMTS) and the Mutualité Sociale Agricole (MSA) databases. In 2004, the CNAMTS insured 83% of the population of the 21 departments under study (from 66% in Gers (SW) to 90% in Meurthe-et-Moselle (NE) and the MSA insured 7% of this population (from 2% in Moselle (NE) to 23% in Gers (SW)). In the CNAMTS and MSA databases, cases were identified as subjects fulfilling at least 1 of the 2 standardized criteria: being registered under the MS official category for long-term illness (internal code Affection de Longue Durée), and being prescribed a treatment (immunomodulatory and/or immunosuppressive) for MS.

MS cases affiliated to the French health insurance scheme for independent workers and other health insurances are presented through the sources ATIH and neurologist network.

Third, we queried the Technical Information Agency of Hospitalization (Agence Technique sur l’Information de l’Hospitalisation- ATIH), which collects all data regarding hospitalizations recorded by the Medical Information Department of all public and private hospitals. We asked the ATIH to provide the number of cases with at least one hospitalization for acute or rehabilitation care, using the International Classification of Diseases-10 code related to MS (G35). All cases of MS recorded had been diagnosed by a medical practitioner or a neurologist.
